# Angulation error assessment for the trajectory in the anteroposterior and lateral fluoroscopic views during percutaneous endoscopic transforaminal lumbar discectomy

**DOI:** 10.1186/s12891-023-06564-x

**Published:** 2023-05-25

**Authors:** Xin Huang, Xiangyu Hou, Shuiqing Li, Bin Zhu, Yan Li, Kaixi Liu, Xiaoguang Liu

**Affiliations:** 1 Pain Medicine Center, Peking University Third Hospital, Beijing, China; 2 Department of Orthopedics, Peking University Third Hospital, Beijing, China; 3 Department of Orthopedics, Beijing Friendship Hospital, Beijing, China

**Keywords:** Percutaneous endoscopic transforaminal discectomy, Angulation error assessment, Trajectory, Cephalad angulation, Virtual fluoroscopy

## Abstract

**Background:**

Anteroposterior (AP) and lateral fluoroscopies are often used to evaluate the intraoperative location and angulation of the trajectory in percutaneous endoscopic transforaminal lumbar discectomy (PETLD). Although the location of the trajectory shown in fluoroscopy is absolutely accurate, the angulation is not always reliable. This study aimed to evaluate the accuracy of the angle shown in the AP and lateral fluoroscopic views.

**Methods:**

A technical study was performed to assess the angulation errors of PETLD trajectories shown in AP and lateral fluoroscopic views. After reconstructing a lumbar CT image, a virtual trajectory was placed into the intervertebral foramen with gradient-changing coronal angulations of the cephalad angle plane (CACAP). For each angulation, virtual AP and lateral fluoroscopies were taken, and the cephalad angles (CA) of the trajectory shown in the AP and lateral fluoroscopic views, which indicated the coronal CA and the sagittal CA, respectively, were measured. The angular relationships among the real CA, CACAP, coronal CA, and sagittal CA were further demonstrated with formulae.

**Results:**

In PETLD, the coronal CA is approximately equal to the real CA, with a small angle difference and percentage error, whereas the sagittal CA shows a rather large angle difference and percentage error.

**Conclusion:**

The AP view is more reliable than the lateral view in determining the CA of the PETLD trajectory.

## Background

Percutaneous endoscopic transforaminal discectomy (PETLD) has become an alternative to conventional open surgery for lumbar disk herniation. An increasing number of surgeons are beginning to learn this technique because of its reliable clinical efficacy [1, 2] and advantages, such as no need for general anaesthesia, fewer cases of iatrogenic neurologic damage, and earlier functional recovery [3, 4]. However, the learning curve for PETLD is steep [5], especially for puncture and localization procedures, which require a whole new way of thinking.

The accuracy of the location and direction of the working channel is crucial in PETLD. Compared with the traditional approach of lumbar discectomy, the trajectory of PETLD requires a longer width skin entry distance from the midline and a larger cephalad angulation (CA), making it very difficult to position and target. An optimal trajectory ensures the successful entry of the cannulated obturator, endoscope, and other relevant instruments into the intervertebral foramen [6], whereas an inaccurate orientation can lead to a higher fluoroscopy frequency, longer surgical time, and an incomplete view, thus increasing the risk of complications [5, 7].

**Fig. 1:**
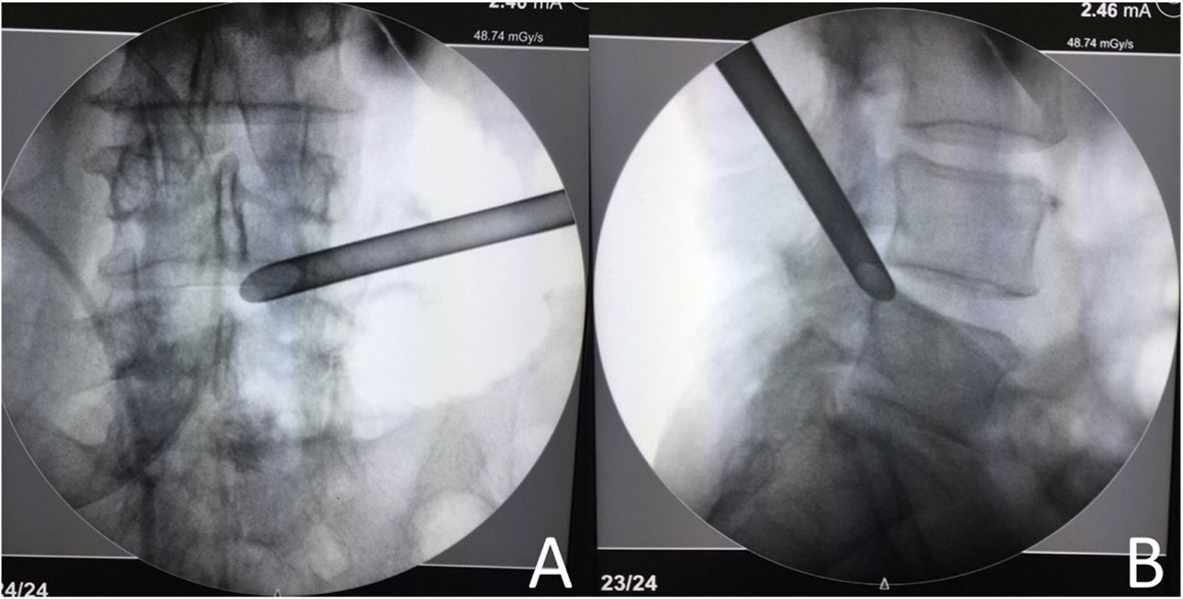
AP (**A**) and lateral (**B**) fluoroscopic views during percutaneous endoscopic transforaminal discectomy. There is often a large difference between the cephalad angulations of the trajectory observed from the AP and lateral fluoroscopic views

Fluoroscopic guidance is a common technique in minimally invasive spine surgeries [8]. Anteroposterior (AP) and lateral fluoroscopies are often performed intraoperatively to evaluate the location and angulation of the trajectory. One problem often encountered during PETLD surgery is that the CAs observed from the AP and lateral fluoroscopic views are often quite different from each other (Fig. 1). Why is that? Which CA is more reliable?

To date, there has been no definitive evaluation method to evaluate the angulation errors in the AP and lateral fluoroscopic views for PETLD in the literature. To solve this problem, a three-dimensional (3D) virtual fluoroscopy method was developed in our study. After reconstructing the computed tomography (CT) images of a human spine, a virtual trajectory was placed into the intervertebral foramen through gradient-changing angulations in reference to the coronal and transverse planes. AP and lateral virtual radiographs were taken and evaluated. Using a formula derivation, the angular relations were demonstrated, and the significance of AP and lateral fluoroscopies was further identified.

## Methods

### Reconstruction of a 3D model

The study protocol was approved by the local institutional review board. Lumbar CT (SOMATOM Definition Flash CT; SIEMENS Healthineers, Erlangen, Germany) with 1 mm slice thickness was performed on one patient with L4-5 herniation. The patient signed an informed consent document for the use of the CT data. Digital imaging and communications in medicine (DICOM) data were acquired and reconstructed with Mimics Research 20.0 software (Materialise, Leuven, Belgium). During the reconstruction, thin-layer CT axial images were first imported into the software. The bone segmentation was completed with the “CT bone segmentation toolkit”. The masks of the lumbar spine, sacrum, and ilium were then specified and reconstructed in a high-quality 3D rendering with the “Calculate 3D” function.

### Virtual trajectory placement

**Fig. 2:**
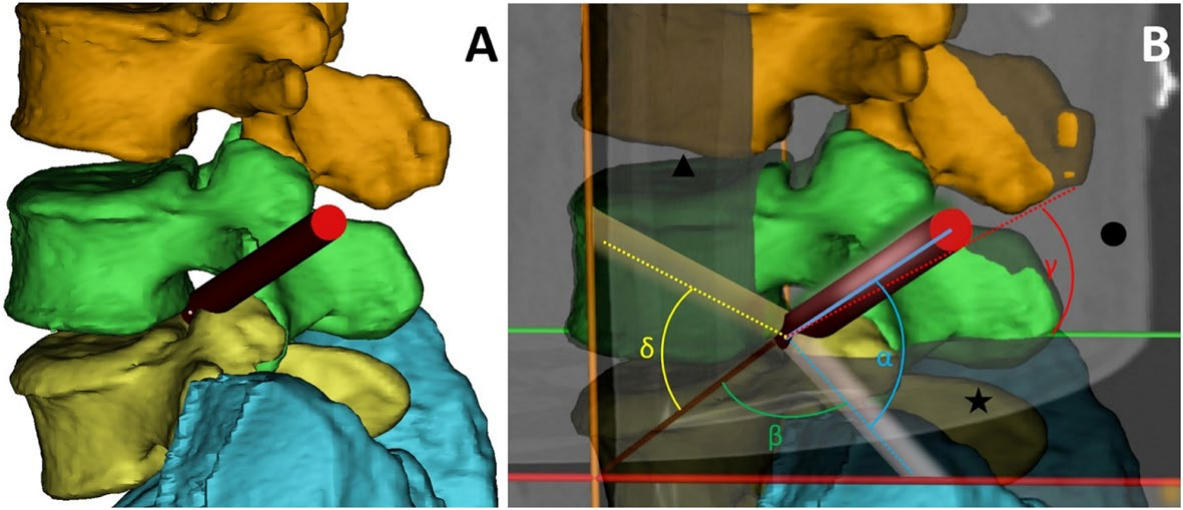
Illustration of the relationship between the virtual trajectory and the anatomic planes. Red cylinder: virtual trajectory; ★: transverse plane; ▲: coronal plane; ●: sagittal plane; α: cephalad angulation (CA), the angle between the virtual trajectory and the transverse plane; β: coronal angulation of the cephalad angle plane (CACAP), the angle between the cephalad angle plane (the plane where the cephalad angle lay) and the coronal plane; γ: the projection of CA on the sagittal plane (sagittal CA); δ: the projection of CA on the coronal plane (coronal CA)

A cylindrical region (radius = 4 mm, length = 150 mm, based on the size of the commonly used cannulated obturator) was established as the virtual trajectory and converted into the Standard Template Library (STL) data format. The cylindrical STL was placed into the spinal canal through the L4-5 or L5-S1 intervertebral foramen, targeting the midpoint between the posterior edges of the two adjacent vertebral bodies in both the transverse and sagittal planes. The relevant anatomic planes and angles are shown in Fig. 2. The transverse plane, coronal plane, and sagittal plane were automatically marked by Mimics Research 20.0 software according to the original CT data.

The cephalad angulation (CA) of the virtual trajectory was defined as the angle between the virtual trajectory and transverse plane; the coronal angulation of the cephalad angle plane (CACAP) was defined as the angle between the cephalad angle plane (the plane where the cephalad angle lay) and the coronal plane; the sagittal CA was defined as the projection of CA on the sagittal plane; and the coronal CA was defined as the projection of CA on the coronal plane (Fig. 2). For the L4-5 level, the CA of the virtual trajectory was set to 10 degrees; for the L5-S1 level, the CA was set to 30 degrees. The virtual trajectory was rotated to a series of predefined angulations in sequence (CACAP: 0°, 15°, 30°, and 45°).

### Virtual fluoroscopy

**Fig. 3:**
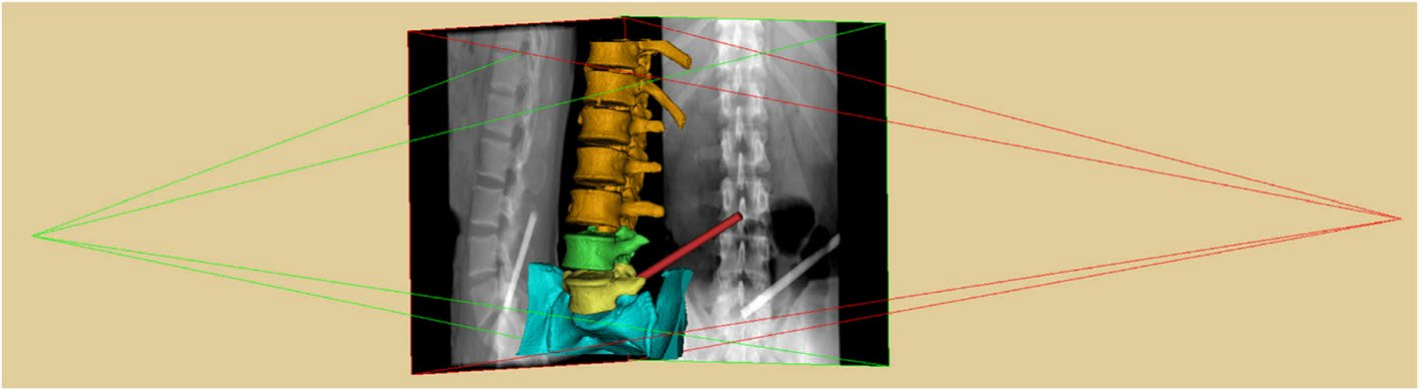
Illustration of the process of virtual fluoroscopy of the lumbar spine and the virtual trajectory

For each angulation, AP and lateral virtual fluoroscopies of the lumbar spine and the virtual trajectory were taken with the “Virtual X-ray Toolkit” of the Mimics Research 20.0 software (Fig. 3). The CAs shown in the AP and lateral views, which indicated the coronal CA and sagittal CA, were measured with the protractor tool of the picpick software (NGWIN, Korea). The angle difference between CA and sagittal (coronal) CA and the percentage error of sagittal (coronal) CA were calculated with the following formulae:

$$\boldsymbol A\boldsymbol n\boldsymbol g\boldsymbol l\boldsymbol e\boldsymbol\;\boldsymbol d\boldsymbol i\boldsymbol f\boldsymbol f\boldsymbol e\boldsymbol r\boldsymbol e\boldsymbol n\boldsymbol c\boldsymbol e\boldsymbol\;\boldsymbol b\boldsymbol e\boldsymbol t\boldsymbol w\boldsymbol e\boldsymbol e\boldsymbol n\boldsymbol\;\boldsymbol C\boldsymbol A\boldsymbol\;\boldsymbol a\boldsymbol n\boldsymbol d\boldsymbol\;\boldsymbol s\boldsymbol a\boldsymbol g\boldsymbol i\boldsymbol t\boldsymbol t\boldsymbol a\boldsymbol l\boldsymbol\;\mathbf{(coronal)}\boldsymbol\;\boldsymbol C\boldsymbol A\boldsymbol=\boldsymbol S\boldsymbol a\boldsymbol g\boldsymbol i\boldsymbol t\boldsymbol t\boldsymbol a\boldsymbol l\boldsymbol\;\mathbf{(coronal)}\boldsymbol\;\boldsymbol C\boldsymbol A\boldsymbol-\boldsymbol C\boldsymbol A$$

$$\boldsymbol P\boldsymbol e\boldsymbol r\boldsymbol c\boldsymbol e\boldsymbol n\boldsymbol t\boldsymbol a\boldsymbol g\boldsymbol e\boldsymbol\;\boldsymbol e\boldsymbol r\boldsymbol r\boldsymbol o\boldsymbol r\boldsymbol\;\boldsymbol o\boldsymbol f\boldsymbol\;\boldsymbol s\boldsymbol a\boldsymbol g\boldsymbol i\boldsymbol t\boldsymbol t\boldsymbol a\boldsymbol l\boldsymbol\;\mathbf{(coronal)}\boldsymbol\;\boldsymbol C\boldsymbol A\boldsymbol=\frac{\mathbf S\mathbf a\mathbf g\mathbf i\mathbf t\mathbf t\mathbf a\mathbf l\boldsymbol\;\mathbf{(coronal)}\boldsymbol\;\mathbf C\mathbf A\boldsymbol-\mathbf C\mathbf A}{\mathbf C\mathbf A}\boldsymbol\times\mathbf{100}\boldsymbol\%$$

### Formula derivation

By means of formula derivation, the angular relations among the real CA, CACAP, coronal CA, and sagittal CA were further verified. The functional curves of the angular relations and the functional curves of the angle differences between the real CA and the sagittal (coronal) CA were drawn. In this part, more CA situations (10°, 30°, 50°, and 70°) were evaluated.

## Results

### Measurement of trajectory angulation

**Fig. 4:**
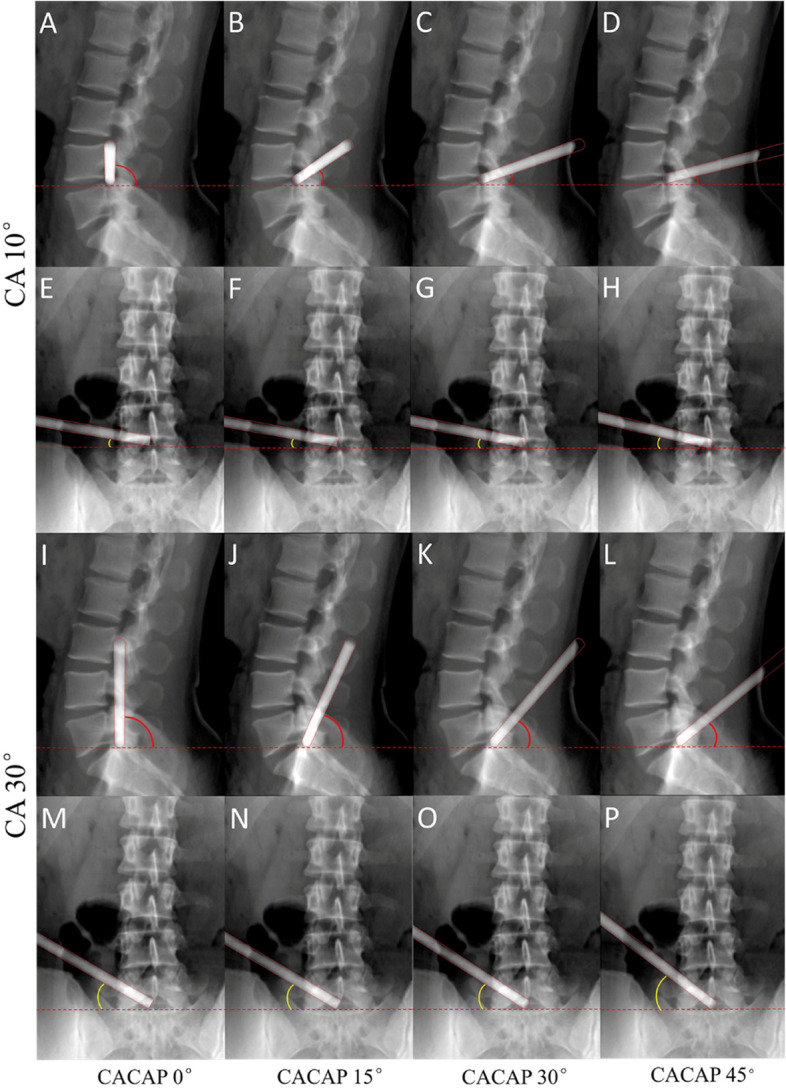
The virtual trajectory was placed into the L4-5 (A-H) and L5-S1 (I-P) intervertebral foramina through a series of predefined angulations. For each angulation, AP and lateral virtual fluoroscopies of the lumbar spine and the virtual trajectory were taken. Lateral: A, B, C, D, I, J, K, and L; AP: E, F, G, H, M, N, O, and P; CA: A-H 10°, I-P 30°; CACAP: A, E, I, and M 0°; B, F, J, and N 15°; C, G, K, and O 30°; D, H, L, and P 45°

The lateral and AP virtual fluoroscopic views of the L4-5 and L5-S1 virtual trajectories are shown in Fig. 4. In the CA 10° and CA 30° groups, the value of sagittal CA decreased markedly with increasing CACAP (Fig. 4A–D, I–L), whereas the value of coronal CA remained almost constant (Fig. 4E–H, M–P).

The measurement of trajectory angulation, the angle difference between CA and its projections, and the percentage error of sagittal (coronal) CA are shown in Table 1. In both the CA 10° and CA 30° groups, with the increment of CACAP from 0° to 45°, the value of the sagittal CA decreased markedly. In the CACAP 0°, 15°, and 30° groups, the angle differences between CA and sagittal CA were much greater than the angle differences between CA and coronal CA, and the percentage errors of sagittal CA were much greater than the percentage errors of coronal CA. When CACAP was not more than 30°, the coronal CA was nearly consistent with the real CA, and the angle differences between CA and coronal CA were quite small, as were the percentage errors of coronal CA.

**Table 1:** Angulations, angle differences, and percentage errors

Variable	Value							
CA (°)	10				30			
CACAP (°)	0	15	30	45	0	15	30	45
Sagittal CA (°)	90.0	34.3	19.4	14.0	90.0	65.9	49.1	39.2
Coronal CA (°)	10.0	10.4	11.5	14.0	30.0	30.9	33.7	39.2
Angle difference between CA and sagittal CA (°)	80.0	24.3	9.4	4.0	60.0	35.9	19.1	9.2
Angle difference between CA and coronal CA (°)	0.0	0.4	1.5	4.0	0.0	0.9	3.7	9.2
Percentage error of sagittal CA	800%	243%	94%	40%	200%	120%	64%	31%
Percentage error of coronal CA	0%	4%	15%	40%	0%	3%	12%	31%

### Angular relations

The angular relations between CA, CACAP, sagittal CA, and coronal CA were derived. If α = CA, β = CACAP, γ = sagittal CA, and δ = coronal CA, then:

$${\varvec{\gamma}}\boldsymbol{ }={\varvec{a}}{\varvec{r}}{\varvec{c}}{\varvec{t}}{\varvec{a}}{\varvec{n}}\frac{\mathbf{tan\,}\boldsymbol{\alpha }}{\mathbf{sin\,}{\varvec{\beta}}}$$

$${\varvec{\delta}}\boldsymbol{ }={\varvec{a}}{\varvec{r}}{\varvec{c}}{\varvec{t}}{\varvec{a}}{\varvec{n}}\frac{\mathbf{tan\,}\boldsymbol{\alpha }}{\mathbf{c}\mathbf{os\,}{\varvec{\beta}}}$$

When β is small (close to 0 degrees), cos β is approximately equal to 1; under such conditions,

$${\varvec{\delta}} \approx {\varvec{a}}{\varvec{r}}{\varvec{c}}{\varvec{t}}{\varvec{a}}{\varvec{n}}\ \mathbf{tan}\ \boldsymbol{\alpha } = \boldsymbol{\alpha }$$

That is, when CACAP is small (the CACAP of the trajectory in PTED is usually small), the coronal CA is approximately equal to the real CA.

### Functional curves

**Fig. 5:**
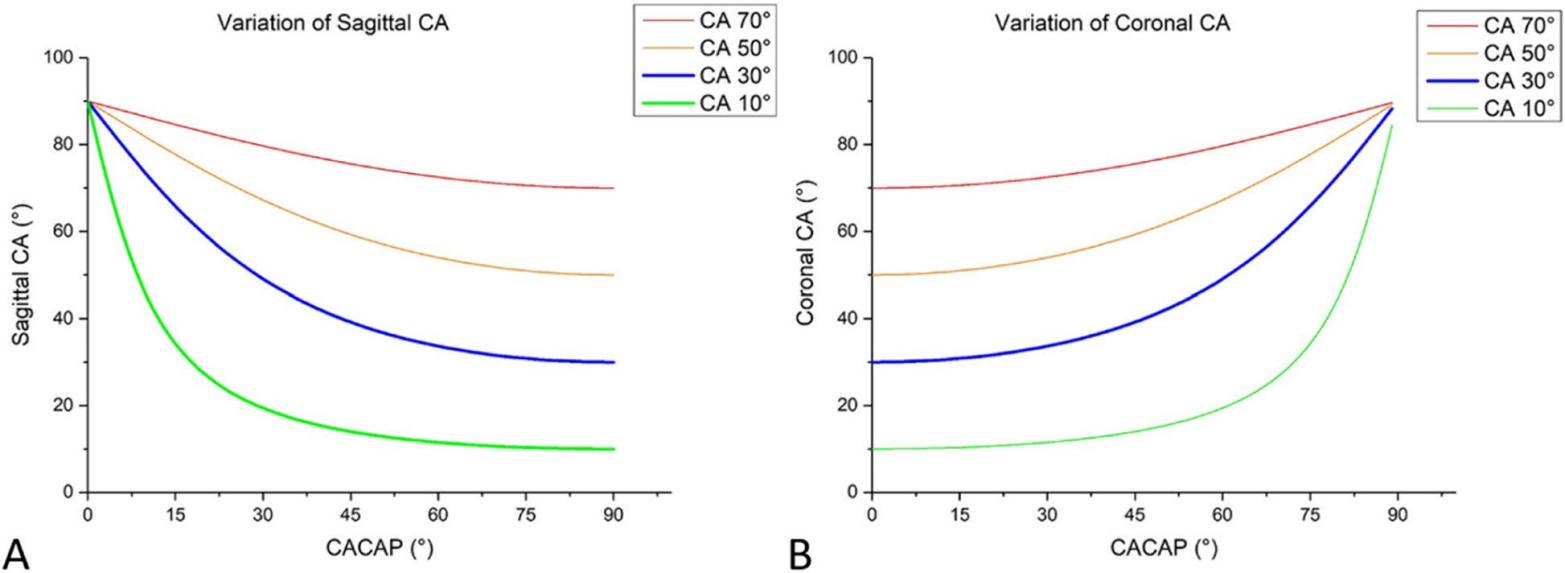
Line graphs showing the variation in the projections of CA on the sagittal plane (**A**) and the coronal plane (**B**)

**Fig. 6:**
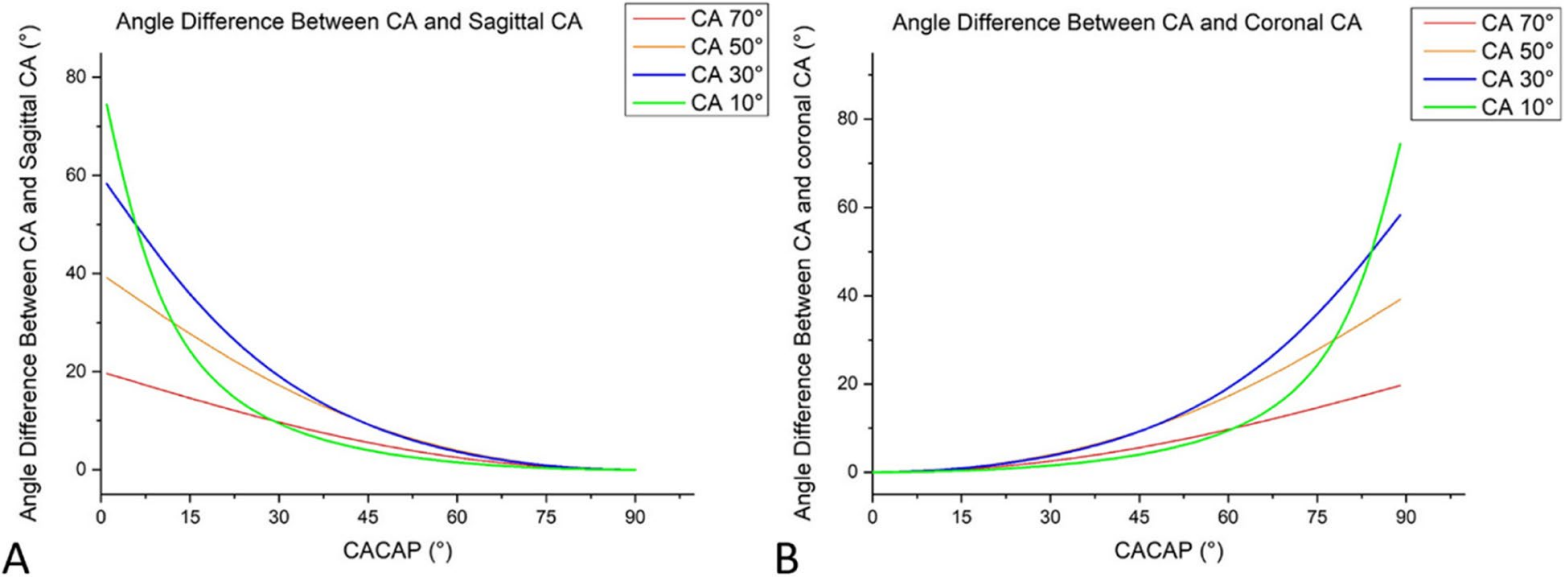
Line graphs showing the angle difference between CA and its projections on the sagittal plane (**A**) and the coronal plane (**B**)

The functional curves of the angular relations are shown in Fig. 5, and the functional curves of the angle differences between CA and its projections on the sagittal plane and the coronal plane are shown in Fig. 6.

When CACAP was less than 30°, the sagittal CA decreased sharply in all CA groups. The smaller CA was, the more sharply the sagittal CA decreased. When CACAP was greater than 60°, the sagittal CA in all CA groups moved gradually and close to the CA baseline (Fig. 5A). When CACAP was less than 30°, the coronal CA moved gradually and was close to the CA baseline in all CA groups. When CACAP was greater than 60°, the coronal CA in all CA groups increased sharply in all CA groups; the smaller CA was, the more sharply the sagittal CA increased (Fig. 5B).

The angle difference functional curves were consistent with the angular relation functional curves. In all CA groups, the angle difference between CA and sagittal CA was apparent when CACAP was less than 30° and became negligible when CACAP was greater than 60° (Fig. 6A). Conversely, the angle difference between CA and coronal CA was negligible when CACAP was less than 30° and became noticable when CACAP was greater than 60° (Fig. 6B).

## Discussion

Traditional posterior lumbar surgery is from the posterior with a skin entry that is a short width distance from the midline, which can directly reach the target with the shortest distance. In this case, the CACAP is large, and the CA is small. For example, the trajectory angulations of percutaneous kyphoplasty and percutaneous pedicle screw fixation are mainly determined by the targeted vertebral pedicle [9]. In the PETLD technique, the working channel enters the spinal canal through the intervertebral foramen; thus, the trajectory of PETLD requires a longer width skin entry distance from the midline (i.e., a small CACAP) and a suitable CA. The requirements of angulations greatly differ among PETLD techniques [4, 10, 11]. In the Yeung endoscopic spine system (YESS) technique, to puncture into the targeted disc and achieve indirect neural decompression, the CA of the trajectory is determined by the targeted disc bisecting the inclination line, and the CACAP of the trajectory is 25° to 30° [10]. In the transforaminal endoscopic spine system (TESSYS) technique, to achieve direct neural decompression inside the spinal canal, the CA of the trajectory is determined with a metal rod that was projected with image guidance towards the isthmus of the upper lamina of each level, and the CACAP differs according to different width skin entry distances from the midline at different levels [4, 11]. The CA in the TESSYS technique is larger than that in the YESS technique. As the entrance point of PETLD is far from the midline, where there is a lack of bony landmarks such as the spinous process, coupled with the different requirements for the different disc levels and the different PETLD techniques, more precise control of the trajectory’s angulation is required in the preoperative localization and puncture procedures of PETLD.

Achieving an optimal CA of the trajectory is important during PETLD surgery. The horizontal approach is a technique for entering the spine without introducing a cephalad angle. In this situation, the CA, sagittal CA, and coronal CA are always 0°. Although the horizontal approach is feasible for segments above L5-S1, our previous work has shown that a suitable CA is more beneficial for PETLD surgery. With an overlarge CA, the trajectory of PETLD may be blocked by some atypical structures of the upper vertebral body, such as the pedicle, transverse process, and accessory process. With an insufficient CA, the trajectory may be blocked by the pedicle and transverse process of the lower vertebral body [12]. For the L5-S1 level, patients may have conditions such as a high iliac crest or lumbosacral abnormality, and the suitable range of CA for the trajectory decreases; thus, more precise control of CA is needed. If the CA is too small, the trajectory may be obstructed by the high iliac crest; if the CA is too large, the excessive angle of the trajectory can make it difficult to pass through the intervertebral foramen and enter the spinal canal [13].

The control of CACAP is mainly determined by the distance from the entrance point to the midline. With an insufficient CACAP, the trajectory may be blocked by the liver, spleen, and kidneys at L1/2 and L2/3 and the intestines at L3/4 and L4/5 [14]. With an overly large CACAP, the trajectory may be blocked by the whole facet joint rather than a ventral part of the superior articular process. For the L5-S1 level with a high iliac crest condition, sometimes it is necessary to enlarge the CACAP and reduce the distance between the entrance point and the midline appropriately to avoid the high iliac crest. Although we can use instruments such as burrs and trephines to enlarge the intervertebral foramen, an optimal angulation of the trajectory may reduce damage to the bony structures.

AP and lateral fluoroscopies are widely used in puncture and localization procedures during surgery. Although new technologies such as the navigation technique [15] and the surgical robot [16] have been applied recently, fluoroscopy remains the most commonly used technique because of its convenience, good economical performance, and considerable effectiveness [17]. AP and lateral fluoroscopy are also commonly used in PETLD to verify the location and angulation of the trajectory. During the puncture process, both AP and lateral fluoroscopy are crucial for determining the location of the trajectory. From the AP view, the distance from the distal end of the trajectory to the lumbar midline can be observed, which aids in determining whether the trajectory has entered the spinal canal; from the lateral view, the depth of the trajectory can be evaluated [18]. If the trajectory is too deep, there is a risk of damage to the abdominal viscera; if it is too shallow, it indicates that the trajectory needs to be adjusted ventrally to reach the intervertebral foramen. During the puncture process, AP and lateral views need to be integrated with each other to adjust the location of the trajectory to reach the target.

During the preoperative localization process, fluoroscopy is crucial for determining the entrance point and angulation of the trajectory. This is because the skin entry point and target (facet joint) determine the final angulation of the trajectory. The location of the trajectory shown in fluoroscopy is absolutely accurate; however, the angulation shown in fluoroscopy is not always reliable. As shown in this study, there is a great difference between the angulations in the AP and lateral views; nevertheless, according to the regularity, we can still evaluate the angulation of the trajectory through intraoperative fluoroscopy. As the trajectory of PETLD requires a longer width skin entry distance from the midline, the CACAP of the PETLD trajectory ranges from approximately 10° to 30°. Under such conditions, the coronal CA is more reliable than the sagittal CA. The coronal CA is approximately equal to the real CA, with a small angle difference and percentage error, whereas the sagittal CA shows a rather great angle difference and percentage error. Because of the high reliability of coronal CA, we can judge the CA according only to the AP view during the preoperative localization procedure in PETLD.

Do the results in this study suggest the CA shown in the lateral view is meaningless? Not truly; the sagittal CA still makes sense. First, it was found that the sagittal CA and coronal CA are always larger than the real CA (Fig. 5), so the real CA can be judged to be small when the CA shown in the lateral view is small. Second, because the sagittal CA is sensitive to the change in CACAP when CACAP is small (Fig. 5), it can be used to detect the change in the patient’s position. As PETLD is performed under local anaesthesia, patients may turn their bodies in case of intraoperative pain caused by inadequate anaesthesia [19], especially in the lateral position, which would change the CACAP (by changing the patient’s anatomic planes) and influence the judgement of the operator regarding the trajectory’s angulation. During surgery, if the trajectory is unaltered while the sagittal CA shows a large change, it is necessary for the operator to reevaluate the patient’s position. Third, CACAP can be roughly estimated from the difference between the sagittal CA and coronal CA. When the sagittal CA is notably larger than the coronal CA, it can be determined that the CACAP is small, which means that the distance between the skin entry point and the lumbar midline is large enough; as CACAP increases to 45°, according to the angular relation formula, the sagittal CA is equal to the coronal CA; when the sagittal CA is smaller than the coronal CA, it can be determined that the CACAP is greater than 45° (Fig. 5). In the latter two cases, it can be interpreted that the entrance point of the trajectory is too close to the midline and should be moved outwards. Moreover, once the intraoperative sagittal CA and coronal CA are measured, the real CA and CACAP can be calculated precisely by the following formulae (derived from the previous formulae):

If α = CA, β = CACAP, γ = sagittal CA, δ = coronal CA, then:

$${\varvec{\beta}} ={\varvec{a}}{\varvec{r}}{\varvec{c}}{\varvec{t}}{\varvec{a}}{\varvec{n}}\frac{\mathbf{tan\,}{\varvec{\delta}}}{\mathbf{tan\,}{\varvec{\gamma}}}$$

$$\boldsymbol{\alpha }\boldsymbol{ }={\varvec{a}}{\varvec{r}}{\varvec{c}}{\varvec{t}}{\varvec{a}}{\varvec{n}}\boldsymbol{ }\left(\mathbf{tan\,}{\varvec{\delta}}\boldsymbol{ }\times \boldsymbol{ }\mathbf{cos}\left({\varvec{a}}{\varvec{r}}{\varvec{c}}{\varvec{t}}{\varvec{a}}{\varvec{n}}\frac{\mathbf{tan\,}{\varvec{\delta}}}{\mathbf{tan\,}{\varvec{\gamma}}}\right)\right)$$

These formulae may be significant for verifying the angulation of the intraoperative trajectory, especially for applications of advanced technologies such as surgical navigation, surgical robotics, virtual reality, and augmented reality surgery systems, which require advanced preoperative trajectory design, intraoperative matching, and registration [20]. The estimation process can provide an important test to compare the intraoperative trajectory with the preoperative designed trajectory, reducing the system error, and improving the system accuracy.

These formulae and regularities are also suitable for the traditional operative approach with a short distance from the midline. It should be noted that the CACAP is large (approximately 70°–90°); in this situation, the sagittal CA is more reliable than the coronal CA.

## Conclusion

In summary, AP and lateral fluoroscopy are commonly used in PETLD to verify the location and angulation of the trajectory. During the puncture process, AP and lateral views need to be integrated with each other to adjust the trajectory location to reach the target. During the preoperative localization process, fluoroscopy is crucial for determining the skin entry point and the angulation of the trajectory. As shown in this study, the AP fluoroscopic view is more reliable than the lateral fluoroscopic view in determining the CA of the PETLD trajectory, whereas the lateral fluoroscopic view still has significance, such as patient position monitoring and CACAP estimation. The angular relations demonstrated in this study may have further use for the application of advanced technologies in PETLD, such as surgical navigation, surgical robotics, artificial intelligence, and augmented reality surgery systems.

## Data Availability

The datasets used and/or analysed during the current study are all included in the article.

## Abbreviations

AP: Anteroposterior
PETLD: Percutaneous endoscopic transforaminal lumbar discectomy
CACAP: Coronal angulations of the cephalad angle plane
CA: Cephalad angles
3D: Three-dimensional
CT: Computed tomography
STL: Standard template library
YESS: Yeung endoscopic spine system
TESSYS: Transforaminal endoscopic spine system
